# Identification and validation of diagnostic signature genes in non-obstructive azoospermia by machine learning

**DOI:** 10.18632/aging.204749

**Published:** 2023-05-24

**Authors:** Lingxiang Ran, Zhixiang Gao, Qiu Chen, Fengmei Cui, Xiaolong Liu, Boxin Xue

**Affiliations:** 1 Department of Urology, The Second Affiliated Hospital of Soochow University, Suzhou, Jiangsu 215004, China; 2 School of Radiation Medicine and Protection, Soochow University, Suzhou, Jiangsu 215123, China

**Keywords:** machine learning, non-obstructive azoospermia, boruta algorithm, lasso regression, LightGBM model

## Abstract

Non-obstructive azoospermia (NOA) is a common cause of male infertility, and no specific diagnostic indicators exist. In this study, we used human testis datasets GSE45885, GSE45887, and GSE108886 from GEO database as training datasets, and screened 6 signature genes (all lowly expressed in the NOA group) using Boruta algorithm and Lasso regression: C12orf54, TSSK6, OR2H1, FER1L5, C9orf153, XKR3. The diagnostic efficacy of the above genes was examined by constructing models with LightGBM algorithm: the AUC (Area Under Curve) of both ROC and Precision-Recall curves for internal validation was 1.0 (*p* < 0.05). For the external validation dataset GSE145467 (human testis), the AUC of its ROC curve was 0.9 and that of its Precision-Recall curve was 0.833 (*p* < 0.05). Next, we confirmed the cellular localization of the above genes using human testis single-cell RNA sequencing dataset GSE149512, which were all located in spermatid. Besides, the downstream regulatory mechanisms of the above genes in spermatid were inferred by GSEA algorithm: C12orf54 may be involved in the repression of E2F-related and MYC-related pathways, TSSK6 and C9orf153 may be involved in the repression of MYC-related pathways, while FER1L5 may be involved in the repression of spermatogenesis pathway. Finally, we constructed a NOA model in mice using X-ray irradiation, and quantitative Real-time PCR results showed that C12orf54, TSSK6, OR2H1, FER1L5, and C9orf153 were all lowly expressed in NOA group. In summary, we have identified novel signature genes of NOA using machine learning methods and complete experimental validation, which will be helpful for its early diagnosis.

## INTRODUCTION

Infertility is a common global health problem; its prevalence was 15%, of which males accounted for 50% [1]. With the changing of lifestyles and the increasing of work pressures, the prevalence of male infertility increased by about 0.291% per year [2]. Abnormal sperm quality was the main manifestation of male infertility, primarily including teratozoospermia, oligozoospermia, and azoospermia, which could exist alone or at the same time. One of the most serious was azoospermia. In addition, depending on whether the ejaculatory duct is obstructed or not, diseases were divided into Obstructive Azoospermia (OA) and Non-Obstructive Azoospermia (NOA). NOA accounted for about 60% of azoospermia and was considered to be one of the most serious types of male infertility [3]. However, for clinical diagnosis of NOA, it can be diagnosed only after the obstruction factors are excluded by physical examination, imaging examination, surgical invasive examination (such as seminal vesicle exploration, testicular biopsy, etc.) NOA could be diagnosed after excluding male reproductive system obstruction. Genetic and chromosome examinations could be improved if necessary to rule out azoospermia caused by genetic factors. At present, the biomarkers with high specificity and sensitivity of NOA were not clear, and there was no clear molecular detection target for NOA, which was disadvantageous to the early diagnosis of NOA. Therefore, finding non-invasive biomarkers, which can reliably predict NOA, can not only eliminate surgical risks, but also reduce the cost of diagnosis and treatment of NOA, resulting in greater benefits for patients.

The machine learning method was to make a preliminary discussion on the possible molecular signal pathway by using bioinformatics technology to comprehensively evaluate multiple databases for modeling [4]. In the era of big data, machine learning methods were widely used in the medical system to provide help for the diagnosis and prognosis of diseases [5]. Especially in the field of imaging and oncology, machine learning has achieved phased results in disease diagnosis, prognosis, and basic research [6, 7]. However, machine learning was rarely used in the early diagnosis of NOA [8].

Therefore, we used machine learning methods to identify new signature genes of NOA and constructed a radiation sterile mouse model [9] to verify the results at the level of pathology and molecular biology. Our results showed that C12orf54, TSSK6, OR2H1, FER1L5 and C9orf153 could be used as indicators for early diagnosis of NOA.

## METHODS

### Data collection

**Figure 1:**
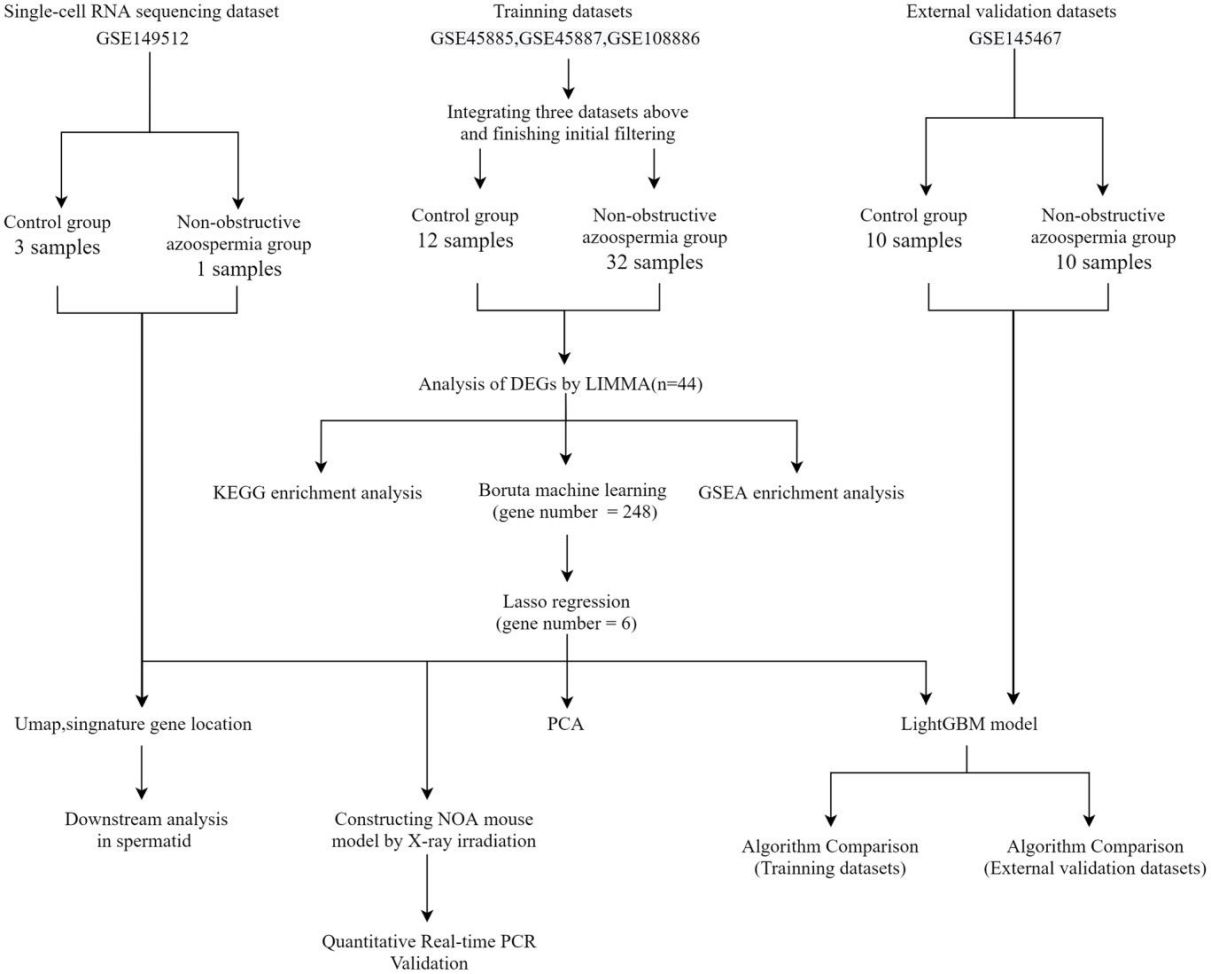
**Flowchart of this study.** Abbreviations: GSE: gene expression omnibus series; LIMMA: linear models for microarray data; DEGs: differentially expressed genes; PCA: principal component analysis.

Figure 1 depicts the study flowchart. Three training datasets (GSE45885, GSE45887, and GSE108886) and one validation dataset (GSE145467) including testis gene expression data for controls and NOA patients, single-cell sequencing dataset (GSE149512) including gene expression data of testis single cell for controls and NOA patients, all were downloaded from the GEO (https://www.ncbi.nlm.nih.gov/geo/) database [10]. Regarding this part, we partially followed the methods of Dr. Zhou et al. (2022) [11].

### Data processing and differentially expressed genes (DEGs) screening

This study was performed in R (version 4.1, https://www.r-project.org/). First, the datasets and clinical information were downloaded using the “GEOquery” package [12]; Second, background calibration, normalization, and log2 transformation on the three training datasets were performed using the “SVA” package in R. When multiple probes of the platform identified one same gene, the average value was calculated to determine its expression. Next, merge the three datasets and eliminate batch effects by using the “SVA” package [13]. Finally, |log2 fold change (logFC)| > 1 and *p*-value < 0.05 were set as the criteria for identifying DEGs using the “Limma” package [14].

### KEGG functional enrichment analysis and gene set enrichment analysis (GSEA)

The Kyoto Encyclopedia of Genes and Genomes (KEGG) is a widely used database for the systematic pathway investigation of DEGs [15]. Gene set enrichment analysis (GSEA) analysis independent of DEGs, including all genes in the analysis to assess relevant pathways and molecular mechanisms [16]. KEGG functional enrichment analysis and GSEA conducted based on the “ClusterProfiler” package [17], |log2 fold change (logFC)| > 1 and *p*-value < 0.05 were set as the criteria for identifying meaningful results.

### Boruta feature selection and lasso regression

After we obtained DEGs, we then performed a preliminary feature screening by the Boruta Feature Selection method [18], a random forest (RF)-based feature filtering machine learning method that helps us select signature genes associated with specific diseases, which was performed using “Boruta” package in R (Arguments: doTrace = 2, maxRuns = 1000, ntree = 500). We then used the least absolute shrinkage and selection operator (LASSO) logistic regression to further filter out most of the genes and included the final results in building the test model [19], which was performed using “glmnet” package in R (Arguments: alpha = 1, family = “binomial”).

### LightGBM model and model performance

LightGBM is a state-of-the-art gradient-boosting framework that uses tree-based learning algorithms [20]. Based on the signature genes obtained by Lasso, we construct LightGBM model on the training datasets using “lightgbm” package in R (the “grid_search” function was used to determine best arguments), and then perform model performance evaluation. Followed by the construction of LightGBM model on the external validation dataset (GSE145467) based on above signature genes and the model performance evaluation. The results are presented as receiver operating curve (ROC) and Precision-Recall Curve.

### Single-cell sequencing dataset analysis

Single-cell sequencing dataset (GSE149512) were downloaded using the “GEOquery” package. We used three control and one NOA patient testis samples to perform analysis. The gene expression matrices were analyzed using the package Seurat (version 4.1.0) [21]. The filtering criteria were set as follows: nFeature_RNA > 200 and nFeature_RNA < 2500 and percent.mt < 20, which means that the cells with detected gene numbers ≤ 200 or gene numbers ≥ 2500 and the proportion of mitochondria ≥ 20% were excluded from the present study. The Top 10 genes exhibiting the most variable among the cells were identified using the “FindVariableFeatures” function. After a series of data integration, filtering, and cleaning, nonlinear dimensionality reduction visualization was performed using the Uniform Manifold Approximation and Projection (UMP) method [22]. Cell clusters were labeled by referring to the original authors' cell markers, to determine the cellular distribution of signature genes [23]. Finally, cells with high signature genes expression abundance were selected using the “subset” function and Spearman’s rank correlation analysis was performed in selected subsets using the “cor.test” function (Argument: type = “spearman”), genes related to signature genes were screened for GSEA pathway analysis to predict possible downstream regulatory mechanisms of signature genes.

### Animal model construction for NOA

Male BALB/c mice aged 6–8 weeks and weighing 20–25 g were purchased from Shanghai Slack laboratory animal Co. Ltd. Animals were kept in Soochow University Animal Center under SPF (Specific Pathogen Free) conditions. Mice handling procedures were reviewed and approved by the Animal Care/User Ethics Committee of Soochow University, Suzhou City, P.R. China. The experimental procedures for all mice were performed in accordance with the Regulations for the Administration of Affairs Concerning Experimental Animals approved by the State Council of the People’s Republic of China.

We used radiation to construct NOA animal models [9], 8 BALB/c male mice were randomly divided into control and irradiated (NOA) groups, and both two groups were sampled on the 21st day after irradiation. The NOA group was irradiated with whole-body 6-MV X-rays at a total dose of 0.5 Gy and a dose rate of 100 cGy/min using a Philips SL18 linear accelerator. The radiation procedure was performed daily with a total of 5 times. After the mice were euthanized, the abdominal cavity was cut open under a stereomicroscope (Olympus, Japan), and the testis and epididymis were carefully separated and photographed. In addition, the testis was weighed using an electronic balance (sartorius, Germany).

### Sperm parameter analysis

The cauda epididymis on one side of the mouse was carefully removed, incised, and incubated in 1 ml Phosphate Buffered Saline (PBS) at 37°C water bath for 5 minutes to allow sperm to release. Gently blow and mix, take 3 μl of the sperm suspension and flush it into the sperm counting plate accompanying the sperm automatic analysis system, put it on the sperm automatic analyzer (BeijingWeili New Century Science and Tech. Co. Ltd., China) for automatic detection, and analyze the sperm concentration and vitality using the WIJY-9000 Sperm Analysis System.

### Sample fixed and HE staining

Testis samples were fixed in modified Davidson’s fluid [24], trimmed, dehydrated, embedded in paraffin, and cut into slices. The slices were dried at 70°C in an oven for 30 min, dewaxed three times in xylene for 5 min each, hydrated in graded concentrations of ethanol at 100%, 95%, 90%, and 85% for 1 min each, stained with hematoxylin for 3 min, treated by ethanol (100%) for 1 min, stained with eosin for 20 s, dehydrated by graded concentrations of ethanol (75%, 80%, 85%, 90%, 95% and 100%) for 1 min each and mounted to slides, followed by fixation with neutral resin. Lastly, the slices were observed under microscopy.

### RNA extraction and quantitative real-time PCR

Total RNA was extracted from testicular tissue using a total RNA extraction kit (Vazyme Biotech Co. Ltd., China), and the reverse transcription process was completed by using the cDNA synthesis kit (Vazyme Biotech Co., China). The cDNA was synthesized by configuring 1 μg of total RNA system according to the manufacturer’s instructions. cDNA was synthesized using the Quantitative Real-time PCR instrument Viia 7 Real-time Polymerase Chain Reaction Detection System from Life Technologies (USA), using SYBR qPCR Master Mix (Vazyme Biotech Co., China) for quantitative polymerase chain reaction. The program was set as follows: after denaturation at 95°C for 2 min, 40 amplification cycles were performed at 95°C for 15 s and 60°C for 32 s. Finally, we normalized the cycle number of target genes and calculated the mRNA expression results. The primer sequences are shown in Table 1.

**Table 1:** Primer sequences in this study.

**Gene**	**Nucleotide Sequence (5′–3′)**
SYCP2-F	GACACTGAAACCGAATGTGGA
SYCP2-R	TGTGGGTCTTGGTTGTCCTTT
SPO11-F	CCCAAGCAGACACACTCCTG
SPO11-R	CTTTTCTGAATGTCCTCTGGCG
PRM3-F	TGGCCTGTGTGAGTCAAGAC
PRM3-R	CTTGCCCTTCACCGGGATTT
TNP1-F	GTCTTCAAACAACACGGGGC
TNP1-R	CGAATTTCGTCACGACTGGC
C12orf54-F	AGCAGCAGAGAAGCACATCC
C12orf54-R	CATTAGCACCTGCTCCCACA
TSSK6-F	TACGTCATGGTCACCGGGTG
TSSK6-R	AACTGTAGCAGCTCGGCGAT
OR2H1-F	TTCGTTGTTGTCTTGTGCTCCTACC
OR2H1-R	CAGGTTGTGGTGAAGCAGAGGTC
FER1L5-F	AAGCTGCCAACTCTGTCTGTGAAG
FER1L5-R	GCTCCACCAGTCCACCTCCTC
C9orf153-F	GCTCTGAGACTGTGAGTGGCAATG
C9orf153-R	TCTGACATGGACGGTGACCTCTG
GAPDH-F	AATGGTGAAGGTCGGTGTGA
GAPDH-R	TGAGTGGAGTCATACTGGAACA

### Statistical analysis

Experiment data were presented as means ± standard deviation $(\bar{x}\;\pm \;\text{SD}).$ GraphPad Prism 8 software was used for statistical analysis, and *t*-test was used for statistical treatment. Data with *P* < 0.05 were considered to be significant.

### Data availability

The data used to support the findings of this study are available from the corresponding author upon request.

## RESULTS

### Identification of differentially expressed genes

**Figure 2:**
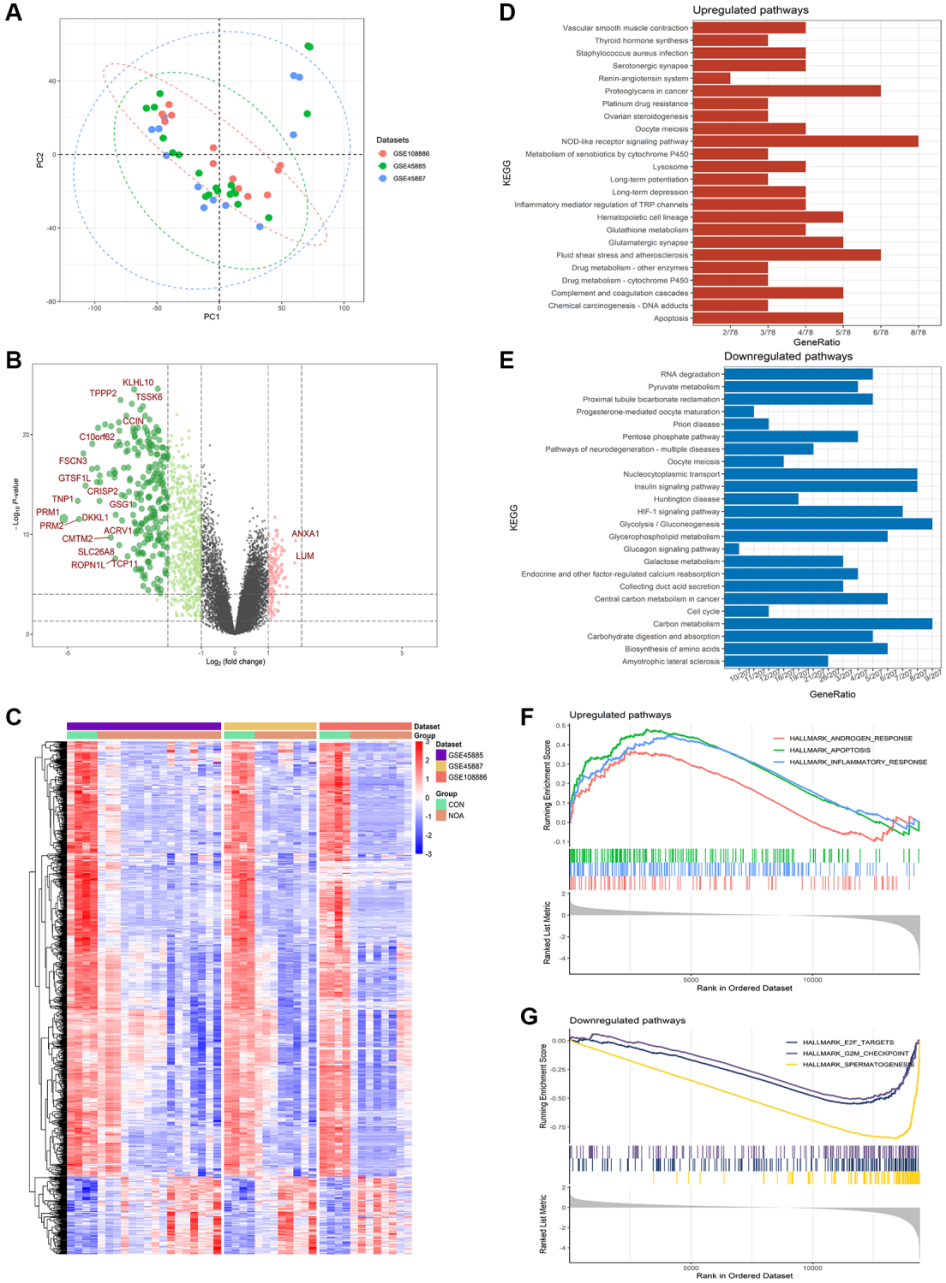
**Differentially expressed genes in NOA versus control.** (**A**) PCA plot of training datasets. (**B**) Volcano plot for the DEGs. (**C**) Heatmap for the DEGs. (**D**) KEGG analysis of upregulated pathways. (**E**) KEGG analysis of downregulated pathways. (**F**) GSEA analysis of upregulated pathways. (**G**) GSEA analysis of downregulated pathways. (*P* < 0.05, Abbreviations: NOA: non-obstructive azoospermia; CON: control group).

As shown in PCA, three sample sources from the training sets were able to cluster well together (Figure 2A). Among the differentially expressed genes in NOA patients compared to control, a total of 942 DEGs were identified in the training combined datasets using the Limma method, of which 143 were upregulated and 799 were downregulated, the volcano and heatmap plot of DEGs are shown in Figure 2B, 2C.

### KEGG functional enrichment analysis and GSEA

KEGG results showed that compared with the control group, DEGs up-regulated in NOA patients were enriched in pathways related to injury factors, such as: “Apoptosis”, “Lysosome”, “Inflammatory regulation of TRP metabolism” and “NOD-like receptor signaling pathway”; while down-regulated DEGs were enriched in pathways related to energy metabolism: “Glycolysis/Gluconemetabolism”, “Glycerophospholipid mediator”, “Pyruvate mediator” (Figure 2D, 2E).

GSEA results showed that compared with the control group, pathways up-regulated in NOA patients were: “Androgen response”, “Apoptosis”, and “Inflammatory response”; down-regulated pathways were: “spermatogenesis”, “G2M checkpoint”, “E2F targets” (Figure 2F, 2G).

### Boruta feature selection and lasso regression

**Figure 3:**
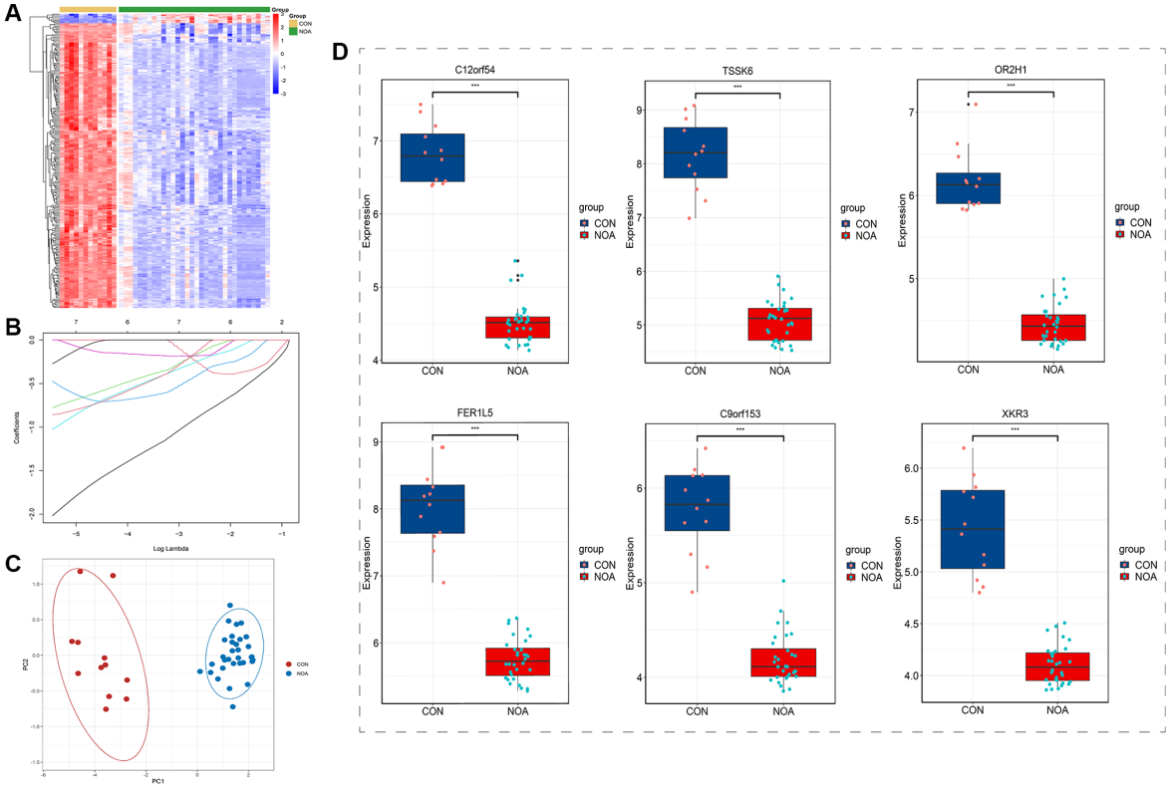
**Boruta feature selection and lasso regression.** (**A**) Heatmap of Boruta screened genes. (**B**) Lasso regression. (**C**) PCA plot of signature genes. (**D**) Expression of signature genes (C12orf54, TSSK6, OR2H1, FER1L5, C9orf153 and XKR3) in training datasets. (*P* < 0.05, Abbreviations: NOA: non-obstructive azoospermia; CON: control group).

After Boruta feature selection screening, 248 signature genes were obtained, and the heatmap plot of signature genes was shown in Figure 3A. After screening by Lasso regression, 6 signature genes were finally left, which were C12orf54, TSSK6, OR2H1, FER1L5, C9orf153, and XKR3 (Figure 3B). PCA results showed that NOA was well separated from controls by these six signature genes alone (Figure 3C). In terms of gene expression, these six signature genes were all underexpressed in NOA patients (Figure 3D).

### LightGBM model and model performance

**Figure 4:**
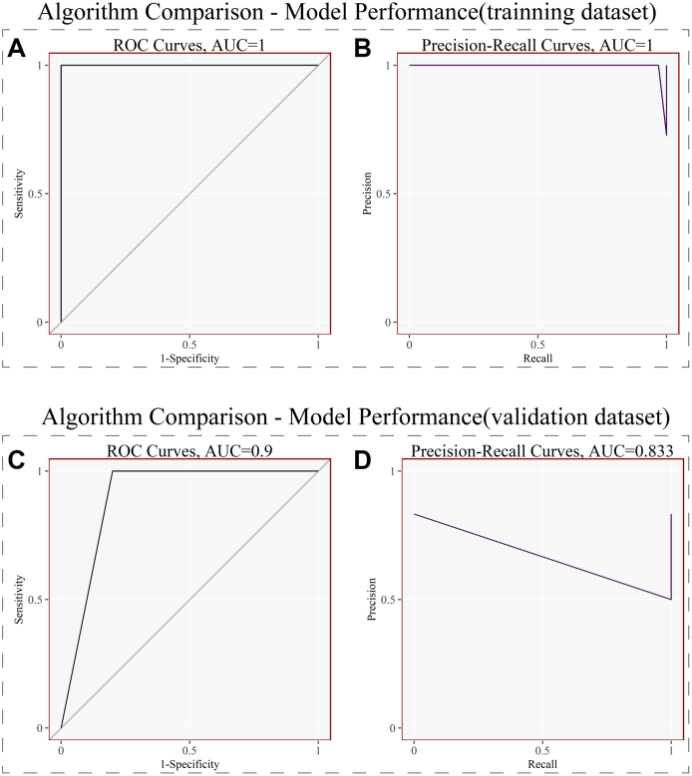
**LightGBM model and model performance.** (**A**) Receiver operating curves (ROC) for signature genes in the training dataset. (**B**) Precision-Recall curves for signature genes in the training dataset. (**C**) Receiver operating curves (ROC) for signature genes in the external validation dataset. (**D**) Precision-Recall curves for signature genes in the external validation dataset. (*P* < 0.05).

We used the above 6 signature genes to construct the LightGBM Model in the training dataset, and the results showed that the AUC (Area Under Curve) of both the ROC and Precision-Recall curves for internal validation was 1.0 (*p* < 0.05) (Figure 4A, 4B). We then used these 6 genes to construct the LightGBM Model on the external validation dataset, and the results showed that the AUC of its ROC curve was 0.9 and that of its Precision-Recall curve was 0.833 (*p* < 0.05) (Figure 4C, 4D). Both internal and external validation illustrated that these 6 genes perform well in diagnostic NOA.

### Single-cell maps determine the cellular location of signature genes

**Figure 5:**
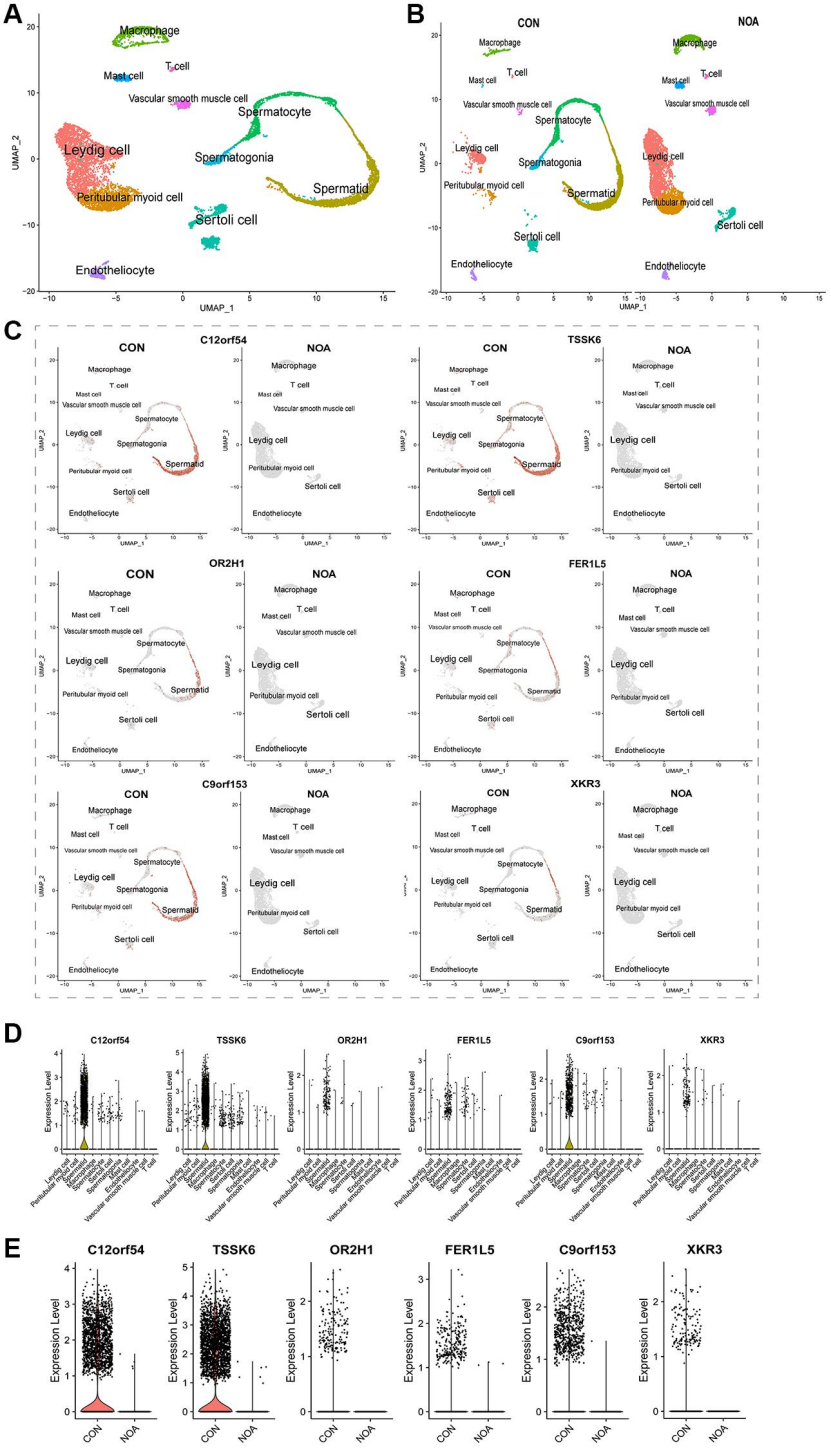
**Single-cell maps define the cellular location of signature genes.** (**A**) Overall UMAP of NOA versus control. (**B**) UMP distinguishing the origin. (**C**) UMAP of cellular localization of signature genes. (**D**) Violin plot of cellular localization of signature genes. (**E**) Violin plot of signature genes expression in NOA versus control. (*P* < 0.05, Abbreviations: NOA: non-obstructive azoospermia; CON: control group).

To determine the cellular localization of these 6 signature genes, we used the Single-cell sequencing dataset GSE149512 for further analysis, the total UMAP and the UMP distinguishing the origin are shown in Figure 5A, 5B. The analysis showed that all 6 genes were expressed in spermatid (Figure 5C, 5D), and they were also lowly expressed in NOA patients (Figure 5C, 5E).

### Downstream regulatory mechanism analysis

**Figure 6:**
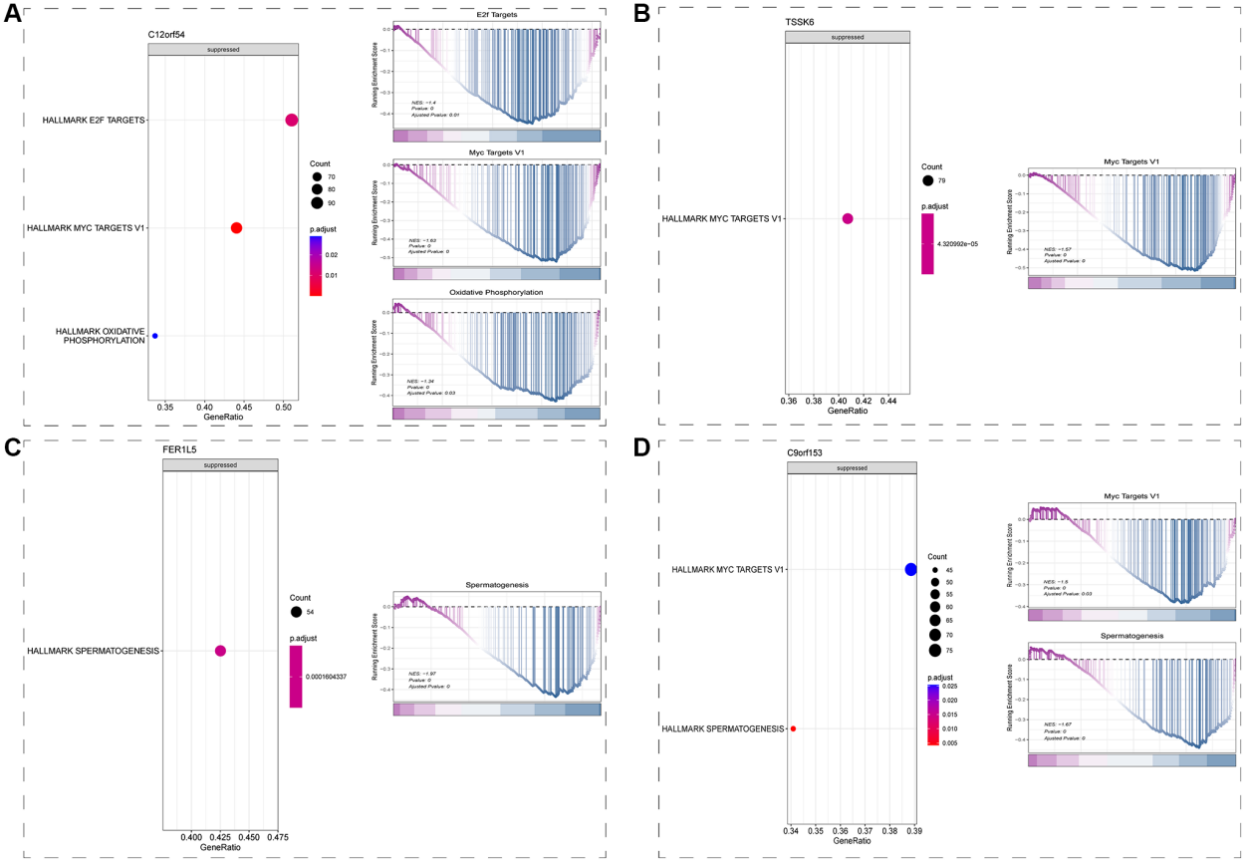
**Downstream regulatory mechanism analysis (GSEA).** (**A**) Downstream regulatory mechanism of C12orf54 in spermatid. (**B**) Downstream regulatory mechanism of TSSK6 in spermatid. (**C**) Downstream regulatory mechanism of FER1L5 in spermatid. (**D**) Downstream regulatory mechanism of C9orf153 in spermatid. (*P* < 0.05).

Having determined that all 6 signature genes were expressed in spermatid, we drew spermatid subsets from the single-cell dataset for their respective downstream regulatory mechanism analysis. Results from GSEA showed that C12orf54 may be involved in the repression of E2F-related, MYC-related pathways and Oxidative phosphorylation-related pathways (Figure 6A); TSSK6 may be involved in the repression of MYC-related pathways (Figure 6B); FER1L5 may be involved in the repression of spermatogenesis pathway (Figure 6C); while C9orf153 may be involved in the repression of MYC-related and spermatogenesis pathway (Figure 6D).

### Animal NOA model construction and experiment validation in testis

**Figure 7:**
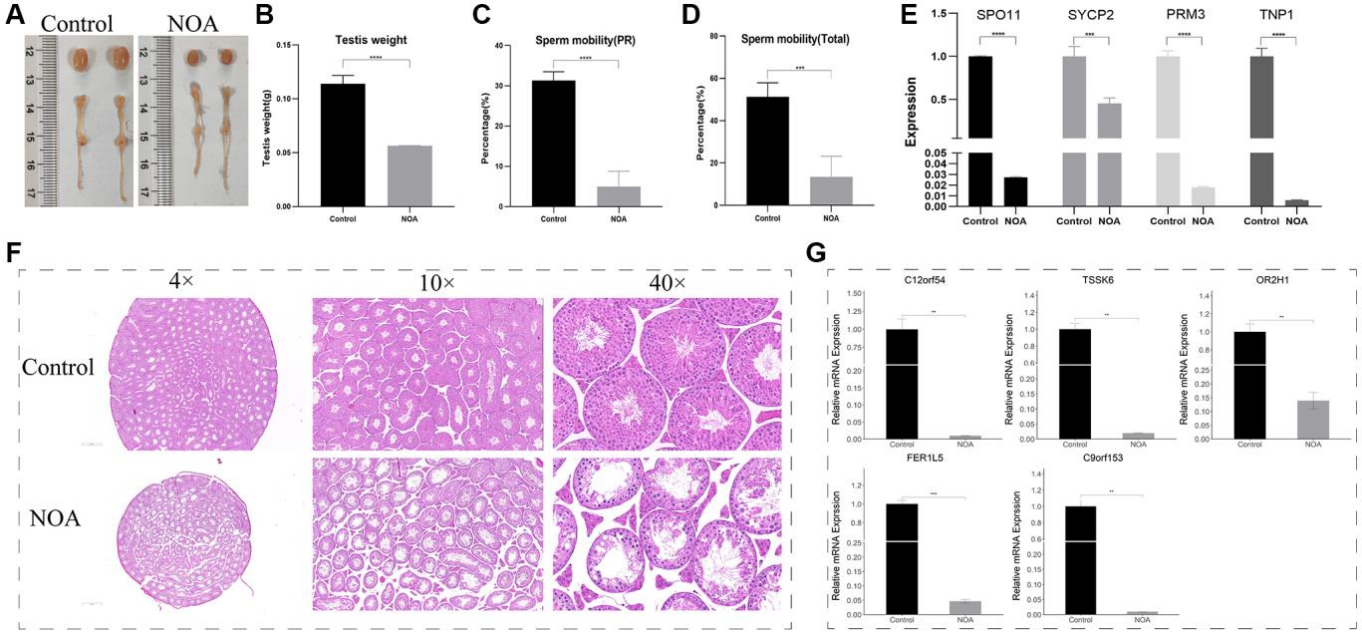
**Animal NOA model construction and experiment validation in testis (NOA: irradiation-induced non-obstructive azoospermia mice, Control: control group).** (**A**) Photographs of testis and epididymis. (**B**) Testis weight (g). (**C**) Sperm progressive motility results (PR). (**D**) Total sperm motility results (Total). (**E**) Relative mRNA expression of SPO11, SYCP2, PRM3, TNP1. (**F**) Testicular HE staining results (4×, 10×, 40×field). (**G**) Relative mRNA expression of signature genes (C12orf54, TSSK6, OR2H1, FER1L5, C9orf153) in testis. (^**^indicates *P* < 0.01, ^***^indicates *P* < 0.001, ^****^indicates *P* < 0.0001).

To confirm the results of our analysis, we constructed the NOA model in mice using X-ray irradiation. The results showed that compared with the control group, the NOA group had severely reduced testicular volume (Figure 7A), severely reduced testicular weight (*p* < 0.05) (Figure 7B), significantly decreased progressive sperm motility and total motility (*p* < 0.05) (Figure 7C, 7D), and significantly decreased mRNA levels of the marker associated with meiosis (*p* < 0.05) (Figure 7E); the testicular HE staining results showed that the spermatogenic function of the testis in irradiated mice was severely damaged (Figure 7F), showed that animal models of NOA were successfully constructed.

Subsequently, in order to detect the differential expression of signature genes in the testis of the two groups, we designed primers to perform quantitative Real-time PCR experiments on the above 5 of 6 signature genes, and the results showed that: C12orf54, TSSK6, OR2H1, FER1L5, and C9orf153 were all lowly expressed in NOA group (Figure 7G).

### Animal experiment validation in epididymis

**Figure 8:**
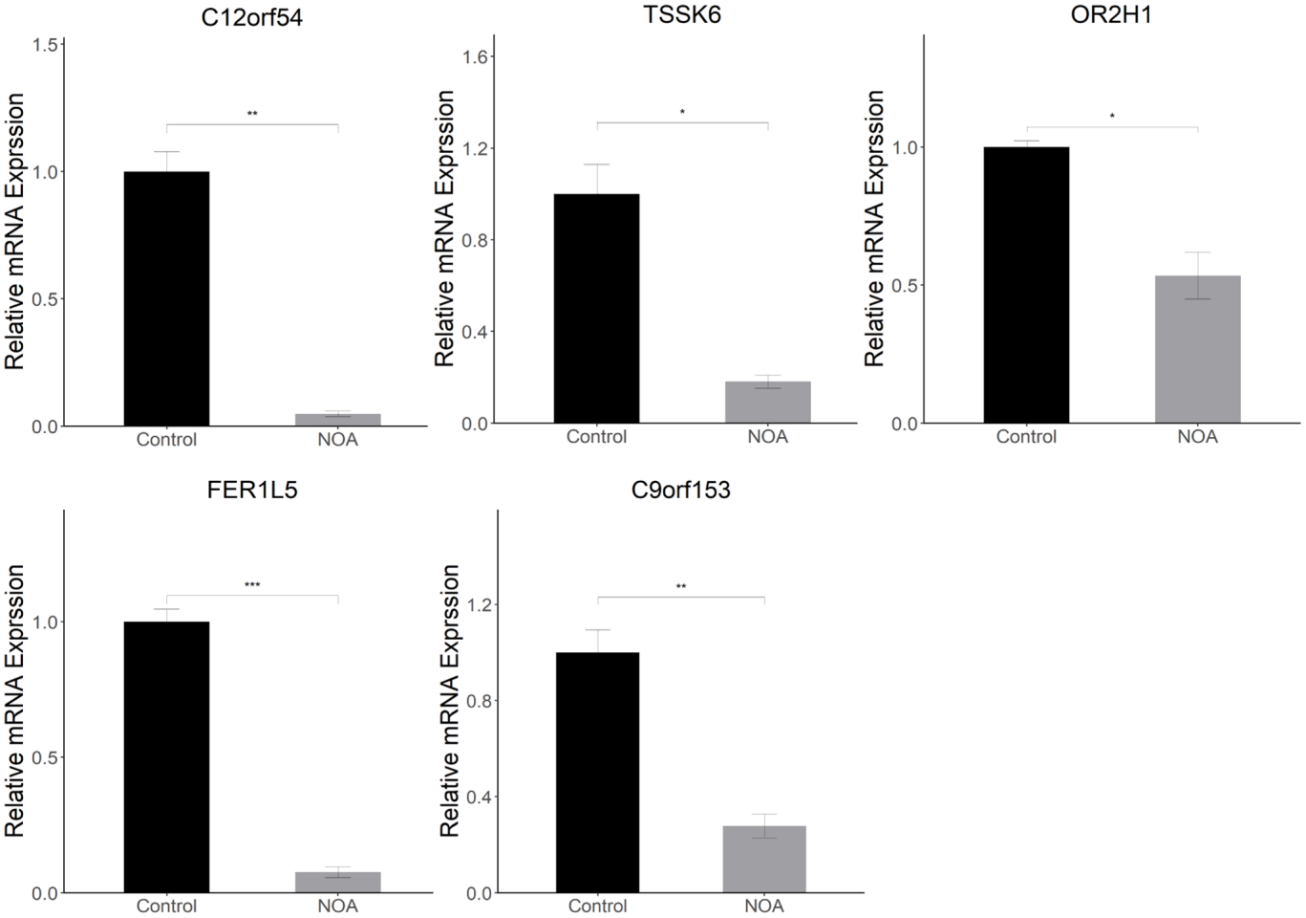
**Animal experiment validation in epididymis (NOA: irradiation-induced non-obstructive azoospermia mice, Control: control group): Relative mRNA expression of signature genes (C12orf54, TSSK6, OR2H1, FER1L5, C9orf153) in epididymis.** (^*^indicates *P* < 0.05, ^**^indicates *P* < 0.01, ^***^indicates *P* < 0.001).

To detect the differential expression of signature genes in the epididymis of the two groups, we performed quantitative Real-time PCR experiments on the above 5 of 6 signature genes using epididymis tissue. Similarly, the signature genes (C12orf54, TSSK6, OR2H1, FER1L5, and C9orf153) were all lowly expressed in NOA group, which showed that the above genes do have potential value in the diagnosis of NOA (Figure 8).

## DISCUSSION

Since Nelson [25] first questioned the changes in male sperm quality, more and more studies have shown that the sperm quality of adult men tends to decline over time [26–29]. In recent years, with the improvement of people's living standards, the problem of male fertility has aroused widespread concern, of which the most complex is azoospermia [30]. This disease affects 1% of men and its causes may include hypothalamic-pituitary dysfunction, primary quantitative spermatogenic disturbances, and urogenital duct obstruction [31]. NOA is a severe idiopathic male factor infertility, which is caused by unexplained disturbance of spermatogenesis, and its mechanism remains to be elucidated [32].

For NOA patients who want to achieve fertility, the only feasible treatment is to obtain sperm by testicular sperm extraction (TESE) and then intracytoplasmic sperm injection (ICSI). Among them, the success rate of sperm extraction in patients with NOA is the focus of attention in the industry. In recent years, several studies have constructed different prediction models to comprehensively evaluate the success rate of TESE in patients with NOA based on the characteristics of genome, transcriptome, proteome, and metabolomics, and screen key molecules to avoid unnecessary TESE surgery [33–35]. However, at present, the related prediction models of NOA are mainly focused on the success rate of sperm extraction and prognosis, while the prediction models for early diagnosis of NOA still lack reliable indicators. In addition, although previous studies have found a number of genes associated with human NOA, the genetic basis of this idiopathic disease is still largely unclear.

Machine learning is a scientific subject that focuses on how computers learn from data, which originates from the intersection of statistics and computer science [4]. In the field of medicine, machine learning is mainly active in collecting and analyzing large datasets related to medical treatment and results, and is expected to transform medicine into a data-driven, results-oriented discipline, which has a far-reaching impact on disease detection, diagnosis, and treatment [5]. There were many methods of machine learning, and our study used the Boruta random forest method, which was a feature selection machine learning method based on random forest (RF), which helped us to select signature genes associated with specific diseases [18]. Because of its excellent feature screening mode, Boruta algorithm has been applied in many fields of medicine, mainly for the screening of signature genes [36–38]. After obtaining the signature gene, we used LightGBM model to construct the prediction model, which is a new gradient-enhanced decision tree algorithm, which has faster training speed, lower memory consumption, better accuracy, and the ability to process large-scale data in order to obtain ideal results [20].

Previous studies have shown that 99.5% of men who receive 12Gy whole-body irradiation have permanent infertility [39], and irradiation dose as low as 5-6Gy can reduce spermatogenesis in convoluted seminiferous tubules in mice [40, 41]. Ionizing radiation can lead to testicular damage leading to prolonged azoospermia and even very low doses of radiation can significantly damage testicular function. Kenta Nagahori and his team have found that ionizing radiation could significantly reduce the weight of the testis, destroy the structure of the testis and affect spermatogenesis by studying the changes of mRNA in mice after single and multiple irradiations, and further results suggested that Sertoli cells played a key role in the immunology of testis induced by radiation [9]. According to previously published results, we successfully constructed the NOA mouse model by fractionated low-dose irradiation [42]. During the 21 days from the end of irradiation to sampling, no obvious abnormality was found in the whole process of the experimental mice, and their body hair, daily behavior, and eating were not affected. Our study not only reduced the effect of high-dose irradiation on the function of organs outside the reproductive system of mice, controlled the confounding factors, and the results were more reliable, but also a kind of animal welfare for the experimental subjects to reduce the unnecessary damage to other important organs caused by radiation.

In this study, we used three datasets of GSE45885, GSE45887, and GSE108886 as training datasets to complete the subject analysis, using testis samples from normal adults and obstructive azoospermia patients with normal spermatogenesis as the control group and NOA patients as the disease group to compare the differences between the two groups. From the PCA results of Figure 2A, it can be seen that the integration of samples from the three datasets was good, and the heatmap of Figure 2C showed that the discrimination of DEGs between the NOA group and the control group of the three datasets was clear, which indicated that this study had high reliability.

Both the volcano plot and heatmap showed us 942 differentially expressed genes in the NOA group compared with the control group (Figure 2B, 2C). Obviously, most of the genes in the NOA group were down-regulated, the most significant of which was the markers of spermatid: PRM1, PRM2, and TNP1(Figure 2B), indicating that their spermatogenic function was seriously affected, which was consistent with our understanding of NOA disease.

In the results of pathway enrichment analysis, KEGG pathway analysis showed that there were pathways related to damage factors such as “Apoptosis”, “Lysosome”, and “Inflammatory regulation of TRP metabolism” in the up-regulated pathways of NOA patients, the down-regulated pathways were mainly related to energy metabolism: “Glycolysis/Gluconemetabolism”, “Glycerophospholipid mediator”, “Pyruvate mediator”, “Cell cycle” and “Biosynthesis of amino acids”. These results suggested that the mechanism of lack of sperm in NOA patients may be related to the inhibition of energy metabolism (Figure 2D, 2E). At the same time, the results of GSEA suggested that the up-regulated pathways also had similar pathways such as “Apoptosis” and “Inflammatoryresponse”, and the down-regulated pathways were “spermatogenesis”, “G2M checkpoint” and “E2F targets”, which indicated that spermatogenesis and cell cycle were inhibited in NOA patients, and may be related to apoptosis and inflammation (Figure 2F, 2G).

After that, we incorporated the obtained DEGs into Boruta feature selection, which was a machine learning method based on random forest. As shown in the heatmap of Figure 3A, after 1000 repeats, we finally selected 248 signature genes that had good discrimination in distinguishing NOA from the control group. Subsequently, we performed Lasso regression on the results of Boruta and finally obtained six signature genes, which were C12orf54, TSSK6, OR2H1, FER1L5, C9orf153, and XKR3 (Figure 3B). From the results of PCA, NOA was well distinguished from the control group by these six genes alone, indicating that they have good potential diagnostic value (Figure 3C). In addition, from the gene expression, these six signature genes were all low expressed in the NOA group, and decreased by a large extent, which was consistent with the results of our heatmap, suggesting that they may be involved in the inhibition of certain pathways.

To further test the diagnostic value of these six signature genes, we built LightGBM Model in the training dataset. After parameter adjustment and correction, the result plots of test efficiency were obtained. As shown in Figure 4A and 4B, the AUC (area under the curve) of the receiver operating curve (ROC) and Precision-Recall Curve in the training dataset were both 1.0 (*P* < 0.05), which indicated that the six signature genes had excellent diagnostic efficiency in the training dataset. To test the results of our analysis, we used GSE145467 as the external validation dataset, and also used these six signature genes to build LightGBM Model. As shown in Figures 4C and 4D, in the validation dataset, the AUC of the ROC was 0.9 and the AUC of the recall curve was 0.833 (*p* < 0.05). The results of both internal and external validation dataset confirmed that the six genes have good potential diagnostic value for NOA.

To determine the cellular localization of these six signature genes, we used testis samples from 3 normal adults and 1 NOA patient from single-cell sequencing dataset GSE149512 for subsequent analysis, and cell clustering was completed with reference to the original author's marker. As shown by the global UMAP, samples from both NOA and the control group could be clearly divided into 11 subpopulations: spermatogonia, spermatocyte, spermatid, peritubular myoid cell, Leydig cell, Sertoli cell, macrophage, T cell, mast cell, vascular smooth muscle cell and endotheliocyte (Figure 5A). From the UMP map of distinguishing sources, we found that all 11 cell groups could be observed in the control group, and mainly spermatogenic cells; while the NOA group lacked spermatogenic cell subpopulations, which also intuitively showed the reason for the lack of sperm in this NOA patient (Figure 5B). Then we observed the cellular distribution of the six signature genes, and the results of the UMP and violin plot showed that they were mainly expressed in spermatids (Figure 5C, 5D). Moreover, all the six signature genes were expressed only in the control group and almost none in the NOA group (Figure 5E). This fully confirmed that these six signature genes were indeed of high potential diagnostic value in NOA.

To explore the possible downstream regulation mechanism of these six signature genes, we extracted data from spermatid subsets according to the results of the previous step, and then completed Spearman correlation analysis between signature genes and other genes to screen genes with correlation *p* < 0.05 and perform GSEA pathway analysis. The results showed that in spermatid, C12orf54 may be involved in the inhibition of the E2F-related pathway, MYC-related pathway, and Oxidative phosphorylation-related pathway (Figure 6A); TSSK6 may be involved in the inhibition of MYC-related pathway (Figure 6B); FER1L5 may be involved in the inhibition of spermatogenesis pathway (Figure 6C); and C9orf153 may be involved in the inhibition of MYC-related pathway and spermatogenesis pathway (Figure 6D). However, OR2H1 and XKR3 were not enriched to a significant pathway, which may be related to their low expression. Among all of possible downstream regulation pathways (all of which showed inhibition), and the most frequent were MYC-related pathways and spermatogenesis pathways. The latter is easily understood because it represents mainly the essential process for sperm production, and the same results emerged in pathway enrichment of Figure 2G. MYC-related pathway, on the other hand, suggests that the pathogenesis of NOA may be related to the inhibition of cell cycle or proliferation. E2F-related pathway may also be a downstream pathway of C12orf54, as well as the G2M checkpoint” and “E2F targets” pathways that also appear in the pathway enrichment of Figure 2G. We suggest that the pathogenesis of NOA may stem from the inhibition of cell proliferation in the testis, which hinders the spermatogenesis process.

Next, in order to confirm the diagnostic efficacy of our six signature genes, we constructed a mouse model of NOA induced by X-ray irradiation [9]. Mice were irradiated with low dose X-ray of 0.5Gy for 5 consecutive days, and sampled on day 21 after the end of irradiation. The results showed that irradiation reduced the testicular volume (Figure 7A), weight (Figure 7B), and sperm motility (Figure 7C, 7D) of mice. Quantitative Real-time PCR of testis tissue and testicular HE pathology results showed that the spermatogenic function of the testis was severely damaged, especially the number of cells in the middle and late stage of meiosis such as pachytene spermatocytes and spermatids decreased significantly (Figure 7D, 7E), which was also consistent with the results of the above single-cell dataset and the training dataset, indicating that we had successfully constructed the mouse model of NOA. Based on this model, we designed primers for the mouse homologous sequences of the above six signature genes, and only five primers were obtained, that were: C12orf54, TSSK6, OR2H1, FER1L5, and C9orf153. Unfortunately, we did not find the mouse homologous sequence of XKR3 and therefore could not design primers for experimental validation. The results of Quantitative Real-time PCR of testis tissue showed that the relative mRNA level of C12orf54, TSSK6, OR2H1, FER1L5, and C9orf153 in the NOA group was lower than those in the Control group, which validated our analysis results in testis.

Finally, in order to better confirm the value of the above signature genes as diagnostic tools for NOA, we performed Quantitative Real-time PCR experiments using epididymis tissue from the control and NOA groups. It is well-established that spermatid produced within the testis are immature and that motility and fertilization ability can only be acquired in the epididymis [43]. At the same time, the cauda epididymis is the storage location of functionally mature spermatids prior to ejaculation, and it stores far more sperm concentration than testis [44]. Therefore, detection of epididymis tissue is also an essential link to evaluate spermatogenic function and can more accurately evaluate the maturation of sperm. Our results showed that in epididymis tissue, the relative mRNA level of C12orf54, TSSK6, OR2H1, FER1L5, and C9orf153 in the NOA group was lower than those in the Control group. The above results in epididymis are consistent with those in testis, which suggests that the above signature genes do have potential value in the diagnosis of NOA.

Further discussion on the translational value of signature genes: (1) At present, the examination of male fertility is mainly based on WHO reference guidelines for semen analysis. That is, computer-aided sperm analysis (CASA) system, which can report the motile percentage and kinematic parameters [45]. However, CASA lacks specificity and efficiency for the diagnosis of NOA. Current diagnostic methods for NOA are more likely to exclusion diagnosis, lack of biomarkers with high specificity and sensitivity. Therefore, we use the Boruta feature selection method to screen diagnostic signature genes based on existing datasets, and the results of analysis show that these genes have high accuracy and can be used as diagnostic markers alone or in combination to construct diagnostic models. The majority of sperm in the epididymis are mature sperm, in which the sperm concentration is also high, so Quantitative Real-time PCR experiment using epididymal tissue can reflect the results of semen sample. In testis and epididymis, the relative mRNA levels of C12orf54, TSSK6, OR2H1, FER1L5, and C9orf153 were significantly lower in the NOA group compared with the control group. Based on the above results, there is rational reason to believe that the signature genes screened in this study have potential value in the diagnosis of NOA and can be developed to clinical application. (2) Diagnostic models based on biomarkers can more economically and efficiently complete the diagnosis of NOA and simplify the standardization of the process by eliminating human bias and reducing workforce to achieve the purpose of rapid diagnosis. The above signature genes can be further used to construct diagnostic models for NOA, which can greatly improve the efficiency of initial diagnosis of NOA, and can even be used as a screening for healthy population. (3) In addition, the construction of diagnostic models may allow stratification of men who are more likely to have successful sperm retrieval prior to the TESE procedure (Despite advances in sperm retrieval techniques, success rates are only 50% [46], and we cannot reliably predict, and therefore cannot predict who may benefit from surgery). The above signature genes can be developed as a stratified indicator in the future for the evaluation of NOA patients prior to TESE to preliminarily distinguish patients who are necessary for surgery and predict the success rate of surgery, which has great clinical significance.

## CONCLUSION

In summary, we have identified novel signature genes of NOA using machine learning methods and complete experimental validation, which will be helpful for its early diagnosis.
